# Smoking, SARS-CoV-2 and COVID-19: A review of reviews considering implications for public health policy and practice

**DOI:** 10.18332/tid/124788

**Published:** 2020-07-03

**Authors:** Emily J. Grundy, Taiba Suddek, Filippos T. Filippidis, Azeem Majeed, Sophie Coronini-Cronberg

**Affiliations:** 1 Chelsea and Westminster Hospital NHS Foundation Trust, London, United Kingdom; 2 Department of Paediatrics, Imperial College Healthcare NHS Trust, London, United Kingdom; 3 Department of Primary Care and Public Health, School of Public Health, Imperial College London, London, United Kingdom

**Keywords:** smoking, public health, coronavirus, COVID-19, SARS-CoV-2

## Abstract

**INTRODUCTION:**

There has been significant speculation regarding the association between the Severe Acute Respiratory Syndrome Coronavirus 2 (SARS-CoV-2) pathogen, coronavirus disease (COVID-19) and smoking. We provide an overview of the available literature regarding the association between smoking, risk of SARS-CoV-2 infection, and risk of severe COVID-19 and poor clinical outcomes, with the aim of informing public health policy and practice, particularly in England.

**METHODS:**

Publications were identified utilising a systematic search approach on PUBMED and Google Scholar. Publications presenting a systematic review or meta-analysis considering the association between smoking and SARS-COV-2 infection or COVID-19 outcomes were included.

**RESULTS:**

Eight studies were identified. One considered the relationship between smoking and the probability of SARS-CoV-2 infection, three considered the association between COVID-19 hospitalisation and smoking history, and six reviewed the association between smoking history and development of severe COVID-19. One study specifically investigated the risk of mortality. The studies considering risk of severe disease indicate that there is a significant association between COVID-19 and current or ever smoking.

**CONCLUSIONS:**

This is a rapidly evolving topic. Current analysis remains limited due to the quality of primary data, although, early results indicate an association between smoking and COVID-19 severity. We highly recommend public health messaging to continue focusing on smoking cessation efforts.

## INTRODUCTION

The current coronavirus disease (COVID-19) pandemic presents a significant public health threat, posing an immediate risk to the health of the global population, and creating far-reaching, long-term consequences. As this new virus spreads, questions continue to emerge regarding risk factors. One widely debated question is the potential effect of smoking on severe acute respiratory syndrome coronavirus 2 (SARS-CoV-2) infection rates and on the clinical outcomes of the resulting disease, COVID-19.

There is well-established evidence that smokers are more susceptible to both viral and bacterial respiratory infections^1,2^, as are those exposed to secondhand smoke^3^. The World Health Organisation (WHO) has also highlighted smokers’ hand-to-mouth action, smoking-induced lung disease, and the sharing of tobacco products such as waterpipes, as factors which may increase a smoker’s vulnerability to SARS-CoV-2 infection and development of COVID-19^4^. Evidence that chronic illnesses, especially respiratory and cardiovascular disease, are risk factors for worse outcomes in COVID-19 is accumulating^5,6^. Smoking contributes to the development of such long-term conditions^2^; conversely, it is reasonable to assume that smoking may increase risks of COVID-19. Early in the pandemic, it was argued that higher mortality among males in China may reflect and be partly explained by the gender disparity in smoking prevalence^7^. Nevertheless, smokers are not currently identified as a vulnerable group within the UK Government’s COVID-19 guidance on social distancing^8^.

There may also be a specific mechanism through which exposure to tobacco smoke can influence infection with SARS-CoV-2. Research following the emergence of severe acute respiratory syndrome coronavirus (SARS-CoV) in 2003 identified a viral binding site on the angiotensin-converting-enzyme 2 receptor (ACE2R)^9^ and it appears SARS-CoV-2 not only utilises ACE2R as its receptor, but may do so more readily than SARS-CoV^10,11^. There are competing theories regarding the effects of smoking on the level of ACER2 expression in cells of the human respiratory tract^10,12,13^. Given the role of ACER2 in enabling viral entry into human cells, any change in ACER2 expression caused by exposure to tobacco smoke (and potentially other nicotine-containing products) may have implications for an individual’s susceptibility to infection.

An opposing theory to this has been proposed, whereby the authors postulate that the nicotinic acetylcholine receptor (nACfR) acts as a co-receptor for viral cell entry within the respiratory tract and central nervous system. It is suggested that nicotine may compete with the SARS-COV-2 for the (nACfR) binding site, hence leading to a reduction in available viral adhesion sites. There is, however, no empirical evidence to support this at this time^14^.

Epidemiological evidence on the role of smoking in COVID-19 is emerging. Considering the wider implications of tobacco control on population health and the healthcare system^2,15^, it is crucial to optimise policies influencing tobacco use during the pandemic. Therefore, we set out to review the findings of existing systematic reviews and meta-analyses regarding the association between smoking and risk of contracting SARS-CoV-2 and of developing severe outcomes once infected, with the intention of informing public health policy, with a particular focus on the public health system in England.

## METHODS

**Figure 1:**
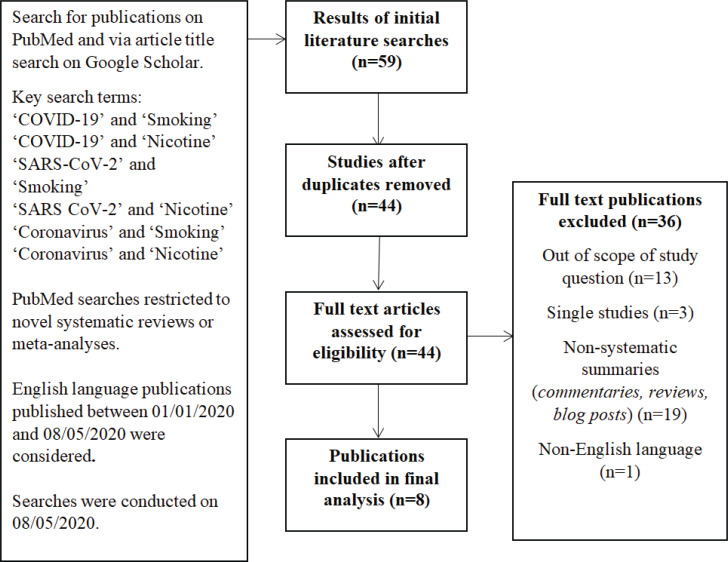
Flow diagram visualising the database searches, number of publications identified, screened, and final full-texts included in this review

A search for existing systematic reviews and meta-analyses published between 1 January and 8 May 2020 was undertaken on PubMed, utilising the search terms: ‘Smoking’, ‘Nicotine’, ‘COVID-19’, ‘SARS-CoV-2’, and ‘Coronavirus’, in pairwise combinations. This was supplemented with an article title search on Google Scholar utilising the same terminology, without restriction on study type, from which meta-analyses and systematic reviews were extracted manually. Two independent researchers screened the publications for eligibility. Only publications in the English language were considered. Our method is explained schematically in Figure 1. In an effort to consider the most up-to-date evidence-base, eligible studies were checked for updates at the point of manuscript revision. The findings from the most recent versions of each study have been considered.

## RESULTS

On reviewing a total of 44 publications, eight studies suitable for inclusion were identified^16-23^, which formed the basis of our review (Figure 1). At the time of writing, three of these eight studies were found to have been updated, or formally published after initially being made available on pre-print platforms^16,18,22^.

### Smoking as a risk factor for SARS-CoV-2 infection

Across the eight selected studies, there were very limited data regarding the relationship between smoking history and risk of SARS-CoV-2 infection. Simons et al.^18^ reviewed 13 studies, using the results from seven of those studies that they deemed ‘fair quality’ to perform a meta-analysis. The results showed that current smokers were at reduced risk of testing positive for COVID-19 compared to never smokers (risk ratio, RR=0.73; 95% CI: 0.73–0.99). There was no significant difference in the risk of testing positive between former smokers and never smokers (RR=1.02; 95% CI: 0.88–1.18). Heterogeneity was very high in both meta-analyses (I^2^=93% and I^2^=84%, respectively).

### Smoking and COVID-19 outcomes

In considering the relationship between smoking and COVID-19 outcomes, the eight studies included in our review took two general approaches:

1. Using data presented in literature to produce pooled smoking prevalence estimates amongst hospitalised patients (hospitalisation being a reasonable proxy for moderate or severe disease)^16-18^;
2. Directly comparing rates of various clinical outcomes among patients hospitalised with COVID-19, according to their smoking history (which is variably defined between studies)^18-23^.

#### Smoking prevalence among hospitalised patients

Three meta-analyses considered hospital admission for COVID-19 and smoking. Two produced pooled estimates of smoking prevalence amongst smokers admitted to hospital with COVID-19^16,17^, whilst the third assessed the risk of hospital admission between smokers, ex-smokers and non-smokers^18^.

Using data for 5960 patients across 13 studies in China, Farsalinos et al.^16^ found a pooled current smoking prevalence estimate of 6.5% (95% CI: 4.9– 8.2%) among hospitalised COVID-19 patients, which was lower than the estimated prevalence within the general population of the country. A secondary analysis designed to address possible misclassification of very recently quit smokers found a pooled estimate of ever smokers of 7.3% (95% CI: 5.7–8.9%). Emami et al.^17^ found a similar pooled estimate of smoking history of 7.63% [95% CI: 3.83–12.43%) based on a smaller sample of 2986 patients derived from six heterogeneous studies from China (I^2^=90.19%). Simons et al.^18^ conducted a rapid review attempting to answer several questions from the available literature. They performed a meta-analysis using five studies deemed ‘fair quality’, showing no difference in either risk of hospital admission between current smokers and never smokers (RR=1.12; 95% CI: 0.74–1.69), or former smokers and never smokers (RR=1.2144; 95% CI: 0.82–1.79).

#### Impact of smoking on disease progression

Six of the studies selected investigated whether smoking was associated with poor clinical outcomes of COVID-19^18-23^. These are described in chronological order of initial publication. Across these studies, the use of the term ‘severe disease’ (also referred to as ‘critical disease’ or ‘disease progression’) was not consistently defined. Three of the studies acknowledged the variability of outcome measures used across the available primary research included within their pooled estimates^18,21,22^, but only two explicitly included detail of how they had defined ‘severe’ disease in the context of their data extraction process and analysis^18,21^. While the Chinese National Health Committee’s definition of severe COVID-19 (‘respiratory distress with respiratory rate ≥30/min or oxygen saturation ≤93% at rest or oxygenation index ≤300 mmHg’)^22^ was used by some of the primary research studies as their outcome of interest, other markers, including ICU admission and mechanical ventilation, or death were also used.

Lippi and Henry^19^ identified five studies from China, incorporating 1399 COVID-19 patients, 288 of whom were identified as having ‘severe disease’. One included study found active smoking to be a significant predictor of COVID-19 severity^24^, but the other four studies showed non-significant results. The pooled odds ratio for the 5 studies was found to be 1.69 (95% CI: 0.41–6.92), although this analysis has been heavily criticised for inclusion of erroneous data^25^.

Vardavas and Nikitara^20^ reviewed outcomes of SARS-CoV-2 in five Chinese studies. In three of the studies reviewed, no significant association could be established between smoking and outcomes by the authors. Results from one study^24^ indicated that smoking was significantly associated with disease progression (OR=14.28; 95% CI: 1.58–25.00; p=0.018). Analysis by Vardavas and Nikitara^20^ using raw data published by Guan et al.^26^, concluded that smokers were more likely to experience severe symptoms (RR=1.40; 95% CI: 0.98–2.00), and 2.4 times more likely to require ICU admission (RR=2.40; 95% CI: 1.43–4.04).

The meta-analysis of Zhao et al.^21^ investigated the risk of development of ‘severe COVID-19’ in COPD patients and current smokers. They included 11 studies with a total of 2002 patients from China. Seven of these studies reported smoking history (n=1726). The pooled results showed an association between current smoking and development of severe COVID-19 (OR=1.98; 95% CI: 1.29–3.05). Zhao et al.^21^ reported moderate study heterogeneity (I^2^=44%; p=0.10). The association between smoking and development of severe COVID-19 was significant when all seven studies were included, however, upon removal of the most heavily-weighted study^26^, which had been found to be a source of heterogeneity, the results were non-significant (OR=1.55; 95% CI: 0.83–2.87). An updated version of this meta-analysis^27^, which included one additional study, showed a stronger association between current smoking and severe disease (OR=2.16; 95% CI: 1.45–3.22), and a reduction in study heterogeneity (I^2^=39%). This result remained significant on the removal of the most heavily-weighted study^26^ from the analyses (OR=1.89; 95% CI: 1.10–3.24)^27^.

Patanavanich and Glantz^22^ published a meta-analysis of 19 studies with their main outcome measure defined as progression of COVID-19 among hospitalised patients. The data analysed included a total of 11590 patients, of which 2133 developed severe disease; 731 patients included were ever smokers. They found that disease progression was observed in 29.8% of ever smokers compared with 17.6% of never smokers (OR=1.91; 95% CI: 1.42–2.59). They reported moderate heterogeneity (I^2^=38%; p=0.048), and no significant publication bias (Harbord’s p=0.813; Peters’ p=0.941). Subsequent sensitivity analysis using five of the 19 studies also found a significant association between current smoking and risk of disease progression compared to never smoking (OR=1.91; 95% CI: 1.10–3.29; p=0.021), with no evidence of significant heterogeneity or publication bias.

Zheng et al.^23^ included five studies from China in their meta-analysis, incorporating data of 1980 patients of whom 268 experienced critical or fatal events. The authors reported a significant association between current smoking and disease progression amongst patients with COVID-19 (OR=2.04; 95% CI: 1.32–3.15). The authors reported no heterogeneity (I^2^=0%).

Simons et al.^18^ reviewed 33 studies investigating the association between disease severity and smoking status. They performed a meta-analysis of four selected ‘fair quality’ studies, showing that current smokers were more likely to develop severe COVID-19 illness compared to never smokers (RR=1.39; 95% CI: 1.09–1.77). No significant difference was observed between former and never smokers (RR=1.40; 95% CI: 0.76–2.59). Simons et al.^18^ treated mortality as a separate measure in their analysis, reviewing 17 studies, and conducting a meta-analysis using two studies deemed ‘fair quality’. These analyses found no significant difference between the risk of death from COVID-19 either between current and never smokers (RR=1.41; 95% CI: 0.91–2.20), or former and never smokers (RR=0.98; 95% CI: 0.65–1.48).

There is some significant overlap between the studies used for the eight systematic reviews and meta-analyses considering: a) smoking prevalence amongst hospitalised patients, b) risk of hospitalisation, c) risk of disease progression, and d) risk of death (Table 1). The total number (n) of included participants in each primary study is also indicated. It should be noted that smoking status was not necessarily available for all participants, and as such, a subset of the total dataset may have been used to inform the authors’ analysis, and this subset may have varied between the eight systematic reviews and meta-analyses.

**Table 1:** Primary studies included by each of the eight systematic reviews or meta-analyses considering: a) smoking prevalence amongst hospitalised patients, b) risk of hospitalisation, c) risk of disease progression, and d) risk of death

*Study (total n)*	*Country*	*Smoking prevalence among hospitalised patients*		*Risk of hospitalisation*	*Risk of disease progression*						

		*Emami et al.*	*Farsalinos et al.*	*Simons et al.*			*Lippi & Henry*	*Vardavas & Nikitara*	*Zhao et al.*	*Patanavanich & Glantz*	*Zheng et al.*

		*2986 [3,A]*	*5960 [1,A]*	*257992 [1,B] 55306 [2,B]*	*1321 [1,C] 1217 [2,C]*	*406 [1,D] 450 [2,D]*	*1399 [1,C]*	*No meta-analysis*	*1726 [1,C]*	*11590 [3,C]*	*1980 [1,C]*
Huang et al. (41)	China	x	x				x	x	x	x	x
Guan, Ni et al. (1099)	China	x	x		x		x	x	x	x	x
Zhang, JJ et al. (140)	China	x	x				x	x	x	x	
Liu et al. (78)	China		x				x	x	x	x	
Yang et al. (52)	China						x		x	x	
Zhou et al. (191)	China		x					x	x	x	x
Chen X et al. (139)	China								x		
Chen T et al. (274)	China		x								
Mo et al. (155)	China		x							x	x
Wan et al. (135)	China		x							x	
CDC (7,162)	USA									x	
Dong et al. (11)	China									x	
Kim et al. (28)	Korea									x	
Shi et al. (487)	China									x	x
Chen N et al. (99)	China	x									
Li et al. (17)	China	x								x	
Liang et al. (1590)	China	x									
Guan, Liang et al. (1590)	China		x								
Lian et al. (788)	China		x								
Jin et al. (651)	China		x								
Guo et al. (187)	China		x								
Zhang X et al. (645)	China		x								
Hadjadj et al. (50)	France				x						
Argenziano et al. (1000)	USA			x							
Miyara et al. (482)	France			x							
Rentsch et al. (3789)	USA			x	x						
Hamer et al. (387109)	UK			x							
Yanover et al. (4353)	Israel			x							
Feuth et al. (28)	Finland				x						
Feng et al. (476)	China									x	
Goyal et al. (393)	USA									x	
Wang et al. (125)	China									x	
Yao et al. (108)	China									x	
Al-Hindawi et al. (31)	UK					x					
Gaibazzi et al. (441)	Italy					x					

Each publication is classified by nature of the question addressed (1=Sample size for effect of current smoking, 2=Sample size for effect of former smoking, 3=Sample size for effect of ever smoking) and outcome of interest (A – rate among already hospitalised patients, B – risk of hospitalisation, C – disease severity, D – death).

## DISCUSSION

We sought to review existing systematic reviews and meta-analyses regarding the association between smoking and risk of both contracting SARS-CoV-2, and COVID-19 disease severity and poor clinical outcomes. We found limited evidence suggesting that the risk of SARS-CoV-2 infection may be lower among smokers compared to non-smokers, albeit from highly heterogeneous studies. By contrast, however, there is growing evidence regarding the association between smoking status and COVID-19 severity and poor clinical outcomes, with an increasing number of published primary research papers making larger, pooled sample sizes possible.

The studies considering risk of severe disease indicate that there is a significant association between COVID-19 and current or ever smoking. The meta-analysis by Patanavanich and Glantz^22^, which included the largest pooled sample size to date, suggests an increased risk of severe COVID-19 amongst ever smokers. While these are important findings, they must be considered in light of the limitations of the primary research.

Other descriptive reviews have acknowledged the contrasting findings presented by Vardavas and Nikitara^20^ and Lippi and Henry^19^ and concluded that early data from China did not provide clear evidence of an association between smoking and poorer COVID-19^7,28,29^ outcomes. However, the methodological soundness of the meta-analysis by Lippi and Henry^19^ has been challenged^25,30^. These criticisms include both the use of incorrect data in meta-analyses^25^ and the incorrect use of null hypothesis significance testing, leading to an incorrect conclusion regarding the absence of effect^30^. A repeat analysis by Guo^25^ using the correct data from the studies considered by Lippi and Henry^19^ found a significant association between active smoking and severe COVID-19 (OR=2.20; 95% CI: 1.31–3.67), supporting the view of Cattaruzza et al.^7^ that despite the uncertainty around data on the association between smoking and COVID-19, smoking may represent ‘the most important avoidable risk factor’. Our paper reviewed a significant amount of the available literature, and hence provides a comprehensive overview of the current evidence-base. It is, to our knowledge, one of the first review of reviews considering the association between SARS-COV-2, COVID-19 and smoking.

### Strengths and limitations

There are several limitations associated with the primary research used to inform the eight systematic reviews and meta-analyses considered within this report. Most of the primary research considered is based on data derived from small convenient hospital samples that are unlikely to be representative of the general population and will under-represent those populations that are unable or less likely to access healthcare.

Furthermore, analysis of those, by definition, non-random samples raises significant risks of selection or collider bias through various pathways. For example, hospitalisation may be influenced both by smoking and COVID-19 severity. Studying hospitalised patients may lead to spurious or mask true associations between smoking and COVID-19 outcomes^31^. Similarly, ex-smokers are also likely to have smoking-related conditions such as cardiovascular illness and pulmonary pathology^2^ making them more vulnerable to severe COVID-19^32^. These smoking-related conditions may in fact be the reason why patients smoke less or quit. Thus, studies investigating current smoking may be subject to selection bias. Investigating the effect of being an ever smoker is less vulnerable to selection bias, though cannot address any hypotheses regarding the direct impact of current nicotine use on disease progression^14^. Some of these issues can be partly addressed with cohort study designs. Two such studies have been conducted in the UK^33,34^ and have found increased risks of hospitalisation and death among current and former smokers. Nevertheless, they also have major limitations, including lack of recent data on smoking status^33^ and unstable estimates in sensitivity analyses^34^.

The primary studies included in the reviews we assessed reported smoking history and outcomes inconsistently. Most studies do not define what is meant by ‘smoking’, and the definitions of current and former smoker are likely to vary. Some report ‘daily smokers’, while others may define former smoker differently, as the duration of abstinence is not clearly standardised. If investigating a correlation between nicotine and COVID-19, reporting of additional nicotine ingestion methods such as vaping, hookah and chewing tobacco should be considered. Definition of ‘severe’ outcomes also varied – often composite measures were used by the meta-analyses to produce a statistically viable sample size.

Recording of smoking status on which many primary studies are based will be of variable quality, particularly at a time when clinical coding may not be a priority. The smoking rates reported are consistently lower than the population averages^18^. However, this should be interpreted with caution because of likely selection bias, but also because smoking prevalence may be substantially underreported leading to misclassification. Several steps in the data acquisition process are prone to systematic error including self-reporting of smoking status, especially at a time when healthcare facilities are under pressure and no next-of-kin may be present to provide further information. Smoking data in health records is often incomplete or inconsistent^35,36^. It is also very challenging to determine how much data may be missing when deriving data from existing datasets and how missing data were treated in each study.

Although most studies referenced by the assessed reviews and meta-analyses are from China, not all are, and this presents another difficulty in comparing datasets across different countries and in assessing generalisability of findings.

Most of the reviews examined utilise data on hospitalised COVID-19 patients. Given that most SARS-CoV-2 infections do not result in medical intervention^37^, the wider picture of the infection pyramid also requires consideration to determine the full picture of how smoking impacts on SARS-CoV-2 infection. Simons et al.^38^ attempted to address this; however, the primary studies available to inform their review were highly heterogeneous, which may decrease the reliability of the pooled estimates.

### Implications for policy and practice

We have reviewed the available international literature considering the association between SARS-CoV-2, COVID-19 and smoking in order to inform public health policy and practice, particularly in England. Although current evidence relating to smoking and COVID-19 is not conclusive, this should not preclude proactive efforts by public health systems to promote smoking prevention initiatives among never smokers, as well as to identify, advise and engage smokers in cessation attempts.

Regardless of any impacts on SARS-CoV-2 infection rates and COVID-19 outcomes, smoking cessation offers a range of benefits, both during the current pandemic and beyond. An obvious example is the reduction in incidence of smoking-related diseases, such as myocardial infarction^39^. Similarly, lower exposure to secondhand smoke can lead to rapid reductions in hospital admissions for asthma exacerbations amongst children^40^ and hospitalisation for myocardial infarction^41^. This latter point is particularly pertinent at this time given the UK Government’s social distancing guidance^8^, which has required people to remain at home for many weeks, potentially changing their smoking practices and leading to an increase in indoor smoking. Delivering these kinds of health improvements in the immediate-to short-term would not only impact on morbidity and mortality rates, but also contribute to a reduction in associated spells of primary, secondary and emergency care during a time in which the NHS is under unprecedented pressure.

The personal financial savings realised as a result of smoking cessation are also significant, particularly for those from deprived communities. Individuals from lower socioeconomic groups are more likely to smoke^42^, and to face increased financial hardship resulting from the impact of lockdown measures and the long-term economic effects.

Survey data suggest that smoking behaviours in England may already have started to shift as a result of the pandemic, though not in wholly positive ways. One survey from April 2020 found that 2% of former smokers surveyed had decided to quit during the previous four months at least in part due to COVID-19. However, in the same survey, 10% of current smokers reported smoking more indoors; 20% buying tobacco in larger quantities and 14% being less likely to try to quit, since the start of ‘lockdown’^43^. COVID-19 may present a ‘teachable moment’ (often associated with health-related events such as surgery, pregnancy, or disease diagnosis)^44^ during which some smokers may be particularly susceptible to smoking cessation messages and motivated to make quit attempts. As such, this opportunity should be harnessed to maximum effect.

Specialist smoking cessation services providing psychological and pharmacological interventions are proven to be cost-effective, and improve success rates compared to unaided quit attempts^45^. On-going reductions to public health funding pose a major challenge in ensuring equitable access to these services, with many local authorities having decommissioned or reduced smoking cessation services provision^46^. Urgent action should be taken to maximise visibility and reach of local, regional and national support that is available, and to enable easy access to pharmacological therapies locally.

Guidance issued by the National Centre for Smoking Cessation and Training aims to support UK smoking cessation services in significantly changing their traditional delivery models in response to COVID-19^47^. This includes increasing capacity for telephone and digital support, facilitating remote access to nicotine replacement products, and pausing the use of carbon monoxide monitoring. While necessary, given current circumstances, these changes present a range of challenges to the effective identification of smokers, verification of self-reported quit attempts and maximisation of successful quit rates, and may contribute to widening health inequalities. Opportunities to share learning around the adoption of new approaches to delivering smoking cessation services should be maximised, including effective messaging to communicate risk around COVID-19 to smokers, and challenging disinformation that may be circulating. Smokers unable to quit should be encouraged to minimise the exposure of household members to secondhand smoke by smoking outside wherever possible, and to practice meticulous hand hygiene to reduce risk of transmission.

At the time of writing, smokers are not designated as high risk from COVID-19 within UK Government guidance^8,48^. This may change as our understanding of a potential association develops. It is important that this relationship be investigated via studies that can address some of the underlying limitations of the existing evidence-base. This should include studies that actively collect (preferably validated) smoking status, rather than relying on existing health records. It also highly important to improve and standardise recording practices regarding smoking status in health and social care settings, to ensure that this cohort is easily identified and offered appropriate guidance.

## CONCLUSIONS

Though early primary research has been constrained by the emerging nature of the COVID-19 pandemic and inherent methodological challenges, more evidence is regularly being published. By considering the findings of eight systematic reviews and meta-analyses, we have conducted a sizeable review of the current evidence regarding the relationship between smoking and COVID-19. We highlight the importance of bespoke research utilising appropriate documentation of smoking status and robust study design. Despite some uncertainty regarding the exact nature and magnitude of the association between smoking and COVID-19, there is growing evidence to support the WHO’s position that *‘smokers are at higher risk of developing severe disease and death’* ^49^. Therefore, public health messaging should strongly highlight the benefits of smoking cessation and not detract from the importance of this during the pandemic or in the aftermath.
